# The Epidermal Growth Factor Receptor (EGFR) Promotes Uptake of Influenza A Viruses (IAV) into Host Cells

**DOI:** 10.1371/journal.ppat.1001099

**Published:** 2010-09-09

**Authors:** Thorsten Eierhoff, Eike R. Hrincius, Ursula Rescher, Stephan Ludwig, Christina Ehrhardt

**Affiliations:** 1 Institute of Molecular Virology, ZMBE, Westfälische-Wilhelms-University, Münster, Germany; 2 Institute of Medical Biochemistry, ZMBE, Westfälische-Wilhelms-University, Münster, Germany; Erasmus Medical Center, Netherlands

## Abstract

Influenza A viruses (IAV) bind to sialic-acids at cellular surfaces and enter cells by using endocytotic routes. There is evidence that this process does not occur constitutively but requires induction of specific cellular signals, including activation of PI3K that promotes virus internalization. This implies engagement of cellular signaling receptors during viral entry. Here, we present first indications for an interplay of IAV with receptor tyrosine kinases (RTKs). As representative RTK family-members the epidermal growth factor receptor (EGFR) and the c-Met receptor were studied. Modulation of expression or activity of both RTKs resulted in altered uptake of IAV, showing that these receptors transmit entry relevant signals upon virus binding. More detailed studies on EGFR function revealed that virus binding lead to clustering of lipid-rafts, suggesting that multivalent binding of IAV to cells induces a signaling platform leading to activation of EGFR and other RTKs that in turn facilitates IAV uptake.

## Author Summary

Obligate intracellular pathogens such as influenza viruses have to enter the host cell to ensure replication and propagation. This process implies the penetration of the plasma membrane. To overcome this membrane barrier viruses have evolved several strategies to abuse the cellular signal-transduction machinery upon binding to specific receptors. Influenza viruses enter the host cell by different endocytotic routes upon attachment to sialic acid residues that serve as direct receptors at the cellular surface. Nevertheless, sialic acids are not able to transmit signals across the plasma membrane, indicating the necessity of additional signaling receptors. In the present study we address the central question, how cells sense influenza virus attachment and how the immediate signaling events upon virus binding are transmitted across the plasma membrane to induce influenza virus internalisation. We show that influenza virus attachment results in a re-arrangement of signaling platforms in the plasma membrane, thereby activating receptor tyrosine kinases that serve as signaling mediators to accomplish virus internalisation.

## Introduction

Influenza A viruses are enveloped viruses that possess a single strand RNA genome with negative orientation. As cell obligate parasites IAV have to enter host cells to ensure efficient replication and propagation by abusing the cellular machinery. Like other enveloped viruses IAV enter the cell by endocytotic routes [1]. The particles are internalized via clathrin-mediated or clathrin and calveolin-independent endocytosis [2]. IAV binds to sialic acid residues at the host cell surface via the viral glycoprotein hemagglutinin (HA). However, these structures possess no signaling capacity and are, thus, not sufficient to induce host cell signaling, which is required to sort the virus particles into specific endocytotic routes. Therefore, it was hypothesized quite early on that engagement of specific signaling-receptors may be required for efficient viral entry [3]. However, the identity of such receptors remained enigmatic.

Among the signaling components, which are induced upon IAV binding are the protein kinase C (PKC) [4], the extracellular signal-regulated kinase ERK [5] and the phosphatidylinositol-3 kinase (PI3K) [6]. PKC has been implicated to be involved in entry processes of various viral pathogens such as rhabdoviruses, alphaviruses, poxviruses, herpesviruses, and IAV [7], [8]. Upon IAV infection, PKC is activated upon attachment of the viral HA to the cell-surface [4]. Activation of the isoform PKCβII results in sorting of the virus particles into the late endosomes [8]. In addition, activation of PI3K and ERK1/2 has been described during entry of human cytomegalovirus [9], [10]. PI3K activation was not only shown to be crucial for IAV entry [6] but also has been demonstrated to regulate vesicular uptake, and trafficking of Ebola viruses [11]. However, it is still elusive, how signaling events are initiated upon early IAV binding and how these signals are transmitted to mediate PKC, ERK and PI3K activation.

A major mechanism that transforms extracellular signals to intracellular PI3K and ERK1/2 activation is the engagement of receptor tyrosine kinases (RTKs). Among the family of RTKs the group of epidermal growth factor receptors, consisting of four members (EGF-, ErbB2-, ErbB3 and ErbB4-receptors) are well studied (reviewed in [12]). Parallels between sorting pathways for the epidermal growth factor receptor (EGFR) and IAV have been identified. Both use endocytotic mechanisms during internalization and require protein ubiquitinylation to be sorted into the vacuolar protein synthesis machinery [13]. Since it was shown that IAV and EGFR were sorted into the same population of late endosomes [2], we hypothesized that RTKs, such as the EGFR may be involved to induce IAV uptake. In this study we investigated the role of EGFR and c-Met, the receptor of hepatocyte growth factor, as candidate RTKs, in IAV entry processes. Broad inhibition of tyrosine kinases by small molecule inhibitors as well as specific EGFR or c-Met inhibition via siRNA mediated knock-down resulted in reduced virus uptake and subsequently to reduced progeny virus titers. Attachment of IAV resulted in clustering of plasma membrane lipids, comparable to EGF stimulation. Our data suggest that IAV is a multivalent agent that, upon binding to sialic acids, is able to cluster and activate EGFR and other RTKs to form a lipid raft-based signaling platform. This leads to receptor-mediated signaling events, such as PI3K/Akt activation, which enhances IAV uptake.

## Results

### Inhibition of tyrosine kinase activity leads to impaired IAV uptake into cells

**Figure 1:**
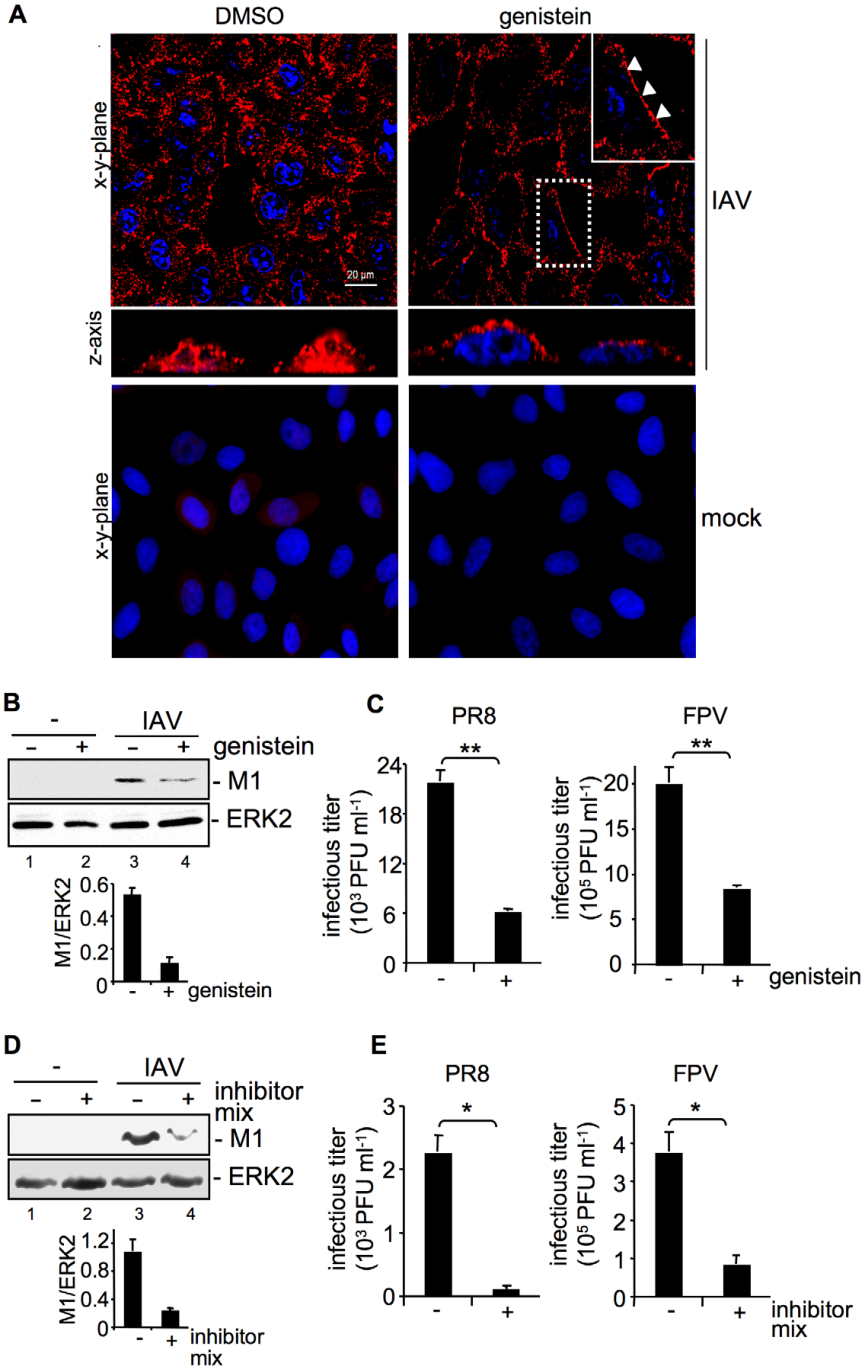
Tyrosine kinase activity is required for efficient uptake of IAV. (A, B, C) A549 cells were treated with genistein (50 µM, [+]) or (D, E) a mixture of several RTK inhibitors (each 10 µM, [+] see experimental procedures) or the solvent control (DMSO,[−]) respectively for 60 min at 37°C or over night prior to infection. (A) Upon genistein treatment, cells were either mock infected or infected with influenza virus A/FPV/Bratislava/79 (H7N7) (FPV; MOI = 100) for further 30 min. Virus particles were visualized in immunofluorescence (IF) microscopy, via a hemagglutinin (HA)-specific rabbit antiserum and an Alexa 594-conjugated chicken anti-rabbit IgG. The nuclei were stained with DAPI. The white arrowheads point to virus particles lining at the cell border. (B, D) After infection with influenza virus A/Puerto-Rico/8/34 (H1N1) PR8 (MOI = 8) for further 30 min, an acidic wash (PBS, pH 1.3, 4°C) was performed before cell-lysis. Internalized virus particles were visualized with a M1 monoclonal antibody in Western-blot (WB) analysis. Equal protein load was verified by ERK2 detection. The relative amount of M1 was quantified (B and D lower panels). Relative M1 densities are expressed as mean ±SD of three independent experiments. (C, E) Cells were infected with FPV or PR8 (MOI = 4) for 8 h; upon the virus-attachment period an acidic wash (PBS-HCl, pH 5.5) was included. Progeny virus titers were determined by standard plaque assays. Data represent mean values of at least three independent experiments ±SD. Statistical significance was assessed by student's t-test (*) p<0.05, (**) p<0.005. See also Figure S1.

To examine the role of RTKs in IAV entry processes, we treated human A549 lung epithelial cells with the broad-range tyrosine kinase inhibitor genistein prior to infection. To detect incoming virus particles cells were infected with a high multiplicity of infection (MOI) and the viral HA was visualized by immunofluorescence (IF) (Figure 1A) as previously described [8]. While in solvent treated cells virus particles were internalized into endosomes 30 min post infection (p.i.) (Figure 1A, upper left panel and z-axis view), in genistein treated cells a completely different staining pattern was observed. A major portion of virus particles appeared to remain stuck to the cell surface (Figure 1A, upper right panels, white arrow heads and z-axis view). To demonstrate the specific inhibitory effect of genistein on the endocytosis of RTKs, we compared the internalization of the EGFR and transferrin (supplementary Figure S1). The EGFR is a representative and ubiquitously expressed family member of RTKs, while transferrin is a glycoprotein, which is internalized independently from RTKs [14]. While transferrin uptake was not affected by genistein treatment, as evidenced by colocalization of transferrin and the early endosomal antigen 1 (EEA1), EGF-induced EGFR internalization was decreased, resulting in a reduced colocalization of EGFR and EEA1 (Figure S1, upper panel). To independently confirm the IF data (Figure 1A), IAV internalization was visualized by detection of virion-associated matrix protein (M1) in Western-blot analysis (Figure 1B) as described by Eierhoff and colleagues [15]. Selective detection of M1 of internalized IAV particles was achieved by applying an acidic washing step before cell lysis to remove attached but not yet internalized virions [15].

M1 protein levels were reduced about 77% in genistein treated cells in comparison to solvent treated cells (Figure 1B) indicating inefficient viral uptake. This correlated with significantly reduced progeny virus titers for the avian virus strain A/FPV/Bratislava/79 (FPV) and the human virus strain A/Puerto Rico/8/34 (PR8) (Figure 1C). The level of titer reduction of about 70% perfectly correlated with the decrease of EGFR internalization with the given concentration of genistein (Figure S1, upper panel). To further confirm these results we also used a RTK inhibitor mix targeting several kinases such as VEGFR, IGFR, PDGFR, EGFR and c-Met. Again virus titers were decreased up to 75% (Figure 1E) concomitant with reduced internalization, as evidenced by 80% lower amount of M1 protein (Figure 1D).

### Increased EGFR abundance enhances IAV uptake

Our results so far indicated that efficient IAV uptake requires, at least in part, tyrosine kinases and probably RTK activity. Based on identification of concurrent endocytotic routes for EGFR and IAV [2], we were prompted to investigate the role of EGFR as a candidate RTK in IAV entry processes.

**Figure 2:**
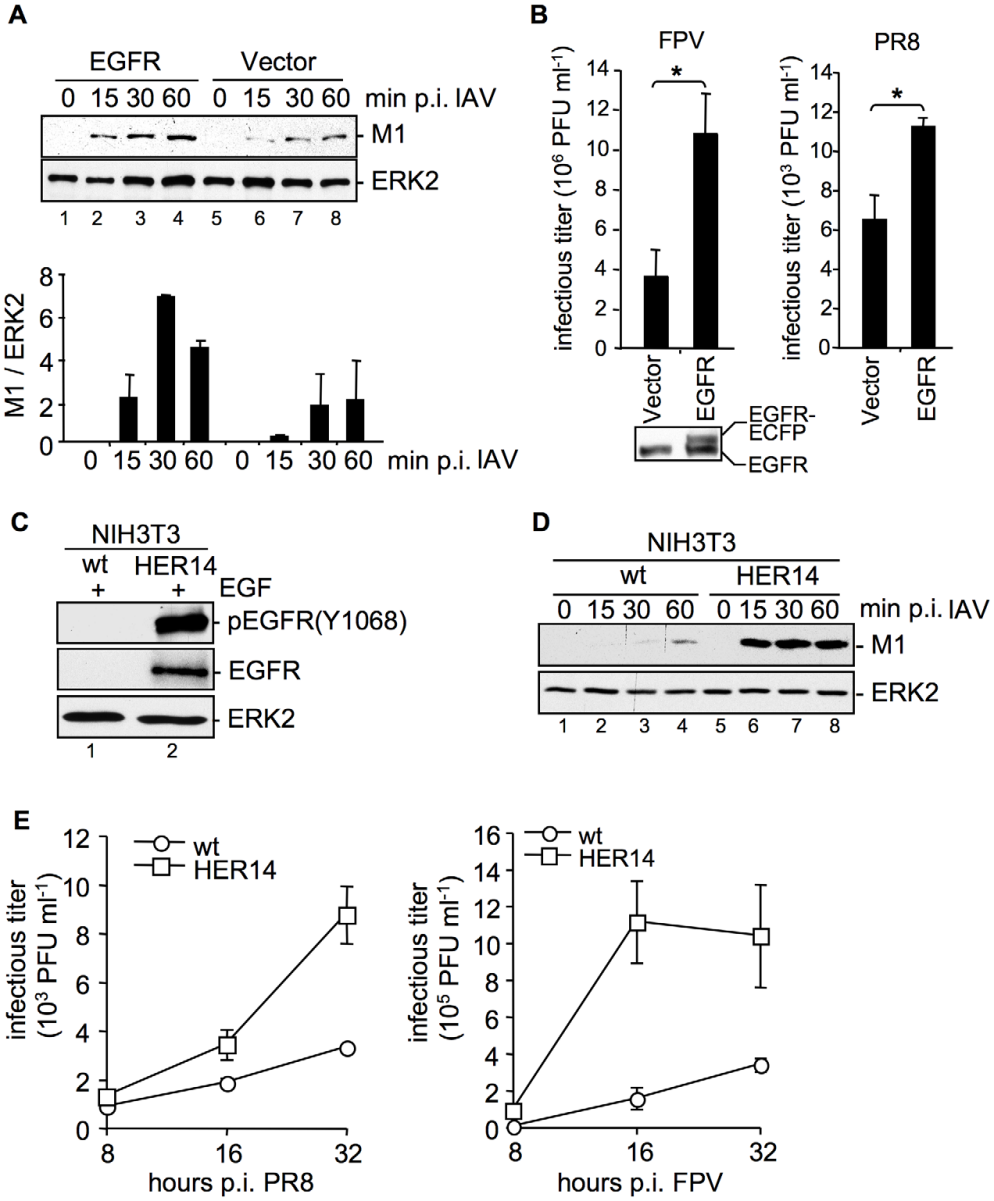
EGFR is involved in IAV uptake. (A, B) An EGFR expressing construct was transfected into A549 cells. (A) After 24 h cells were infected with PR8 (MOI = 4) for the indicated time points. An acidic wash (PBS, pH 1.3, 4°C) was performed before cell-lysis to remove not internalized virus particles. In WB analysis M1 and ERK2 were detected. The relative amount of M1 was quantified (A, lower panel). Relative M1 densities are expressed as mean ±SD of three independent experiments. (B) Cells were infected with FPV (MOI = 0.5, left panel) or PR8 (MOI = 0.1, right panel) for 8 h; upon the attachment period an acidic wash (PBS-HCl, pH 5.5) was included. Progeny virus titers were determined by standard plaque assays. Data represent mean values of at least three independent experiments ±SD. Statistical significance was assessed by student's t-test (*) p<0.05. In WB analysis EGFR expression level of uninfected cells was detected. (C, D) NIH3T3 wild-type (wt) and NIH3T3 EGFR expressing (HER14) cells were incubated with (C) EGF (30 ng ml^−1^, 10 min, 37°C) or (D) were infected with PR8 (MOI = 4) for the indicated times. Upon infection an acidic wash (PBS, pH 1.3, 4°C) was performed before cell-lysis. WBs were probed with the indicated antibodies (pEGFR (Y1068) is the autophosphorylation site in the EGFR at tyrosine 1068). (E) NIH3T3 wild-type (wt) and NIH3T3-Her14 (HER14) cells were infected with PR8 and FPV (MOI = 1) for the indicated times. Upon the attachment period an acidic wash (PBS-HCl, pH 5.5) was included. Progeny virus titers were determined by standard plaque assays. Data represent mean values of three independent experiments ±SD. See also Figure S2.

Cells were transfected with a tagged form of the EGFR. Albeit only a moderate expression of the exogenous EGFR was detectable (Figure 2B), these enhanced levels of the receptor were sufficient to result in a significant increase of virion-associated M1 protein 15 min to 60 min p.i., in comparison to vector transfected cells (Figure 2A). This indicates accelerated virus internalization in the presence of increasing amounts of EGFR. Again, enhanced IAV internalization correlated well with an increase in virus yields from cells over-expressing EGFR (Figure 2B). To further confirm an involvement of EGFR in IAV uptake, we employed the mouse embryonic fibroblast cell line NIH3T3 that does not express endogenous EGFR and the NIH3T3-Her14 cell line stably expressing EGFR [16]. Functionality of the NIH3T3-Her14 cell line was verified in Western-blot analysis: only in NIH3T3-Her14 cells, expression of a functional EGFR that was activated by the ligand was detectable (Figure 2C). While upon infection of control cells virus-associated M1 protein was barely detectable, a strong increase of M1 levels was observed in Her14 expressing cells, indicative of strongly enhanced virus uptake (Figure 2D). Further, NIH3T3 wt and NIH3T3-Her14 cells were infected with the IAV strains PR8 and FPV and progeny virus titers were monitored over a time period of 32 hours (Figure 2E). IAV replicated well in both cell types, however, in NIH3T3-Her14 cells virus titers were enhanced at every time point analysed. This observation strengthens the previous findings that virus internalisation was more efficient in EGFR expressing NIH3T3 cells.

### Impaired expression, or activation of EGFR and c-Met results in reduced internalization of IAV

**Figure 3:**
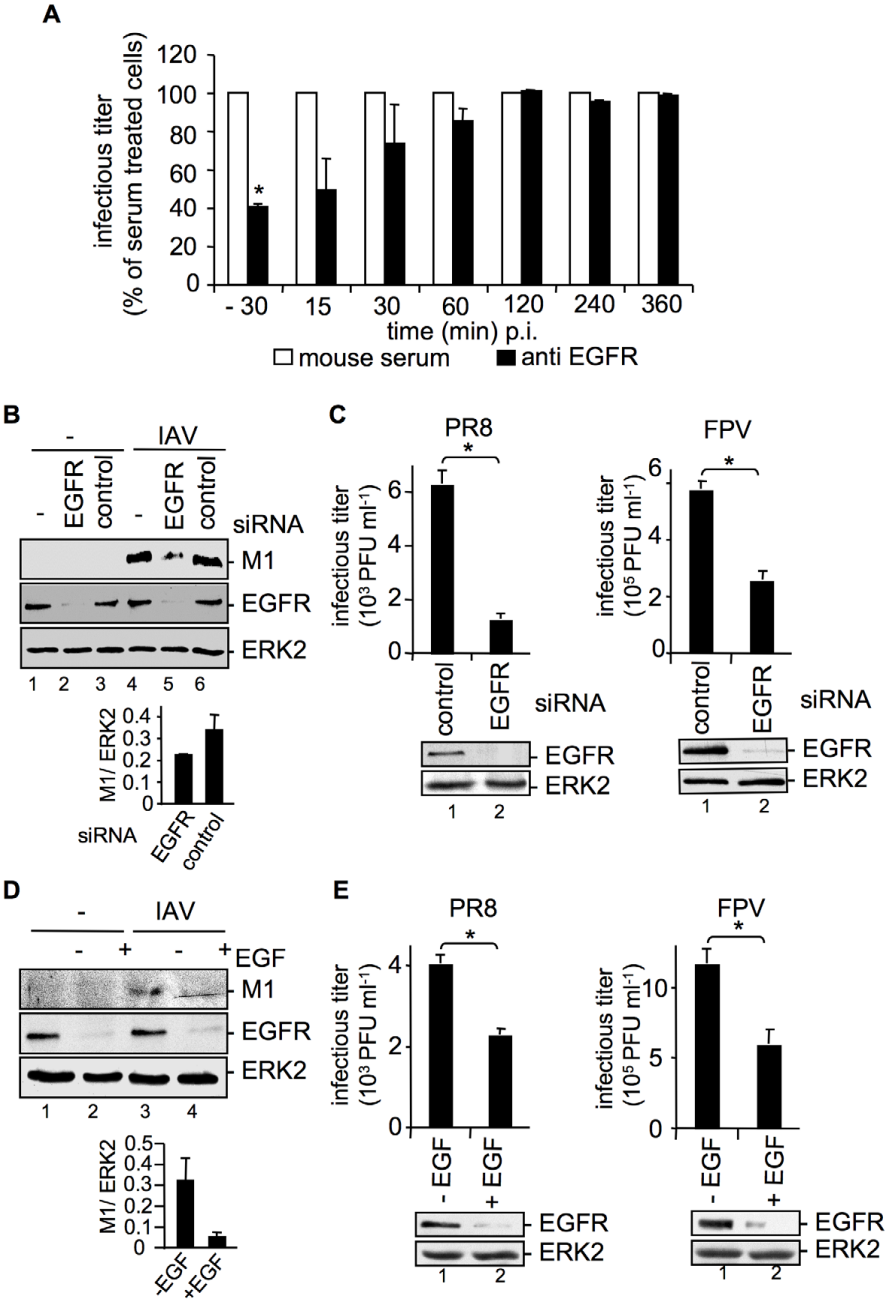
EGFR knock-down impairs efficient uptake of IAV into A549 cells. (A) A549 cells were treated with an EGFR-specific antibody (5 µg ml^−1^) or mouse serum at the indicated times before and during infection with FPV (MOI = 0.5) for 8 h. Progeny virus titers were determined by standard plaque assays; virus yields of serum treated cells were arbitrarily set as 100%. Calculation of virus titers revealed 5.65×10^5^ PFU ml^−1^ (±1.2×10^5^) for -30 min pre-incubation, 10×10^5^ PFU ml^−1^ (±1×10^5^) for anti EGFR- and for serum pre-incubation. Statistical significance was assessed by student's t-test (*) p<0.05. (B, C) A549 cells were transfected with a control siRNA or a specific siRNA targeting the EGFR. 48 h post transfection cells were infected with (B) PR8 (MOI = 4) for 30 min or (C) PR8 or FPV (MOI = 4) for 8 h. (C) Upon the attachment period an acidic wash (PBS-HCl, pH 5.5) was included. (D, E) A549 cells were incubated with EGF (100 ng ml^−1^) 90 min before infection with (D) PR8 (MOI = 4) for 30 min or (E) PR8 or FPV (MOI = 4) for 8 h. (B, D) Before cell-lysis an acidic wash (PBS-HCl, pH 1.3, 4°C) was performed and cell-lysates were analysed in WB. Relative M1 densities are expressed as mean ±SD of three independent experiments. (C, E) Progeny virus yields were determined by standard plaque assays. Data represent mean values of three independent experiments ±SD. Statistical significance was assessed by student's t-test (*) p<0.05. EGFR knock-down was verified by WB before infection (C and E, lower panels). Cells were transiently transfected with an EGFR construct during RNAi mediated knock-down (B) or 24 h prior to EGF induced EGFR degradation (D). See also Figure S1 and S2.

So far our results indicated that over-expression of the EGFR enhances early IAV uptake. To further foster these findings, an EGFR blocking antibody was employed at different time points pre- and post infection. The results showed that only when EGFR was inhibited at early stages during virus infection, progeny virus titers were significantly reduced (Figure 3A). Inhibition of EGFR 30 min p.i., or later showed no effects anymore. These findings were further supported by an approach using EGFR specific siRNA. Efficient EGFR knock-down was confirmed in Western-blot analysis (Figure 3B and 3C). Down-regulation of EGFR resulted in reduced internalization of virus particles as evidenced by reduced levels of virion-associated M1 protein in cells (Figure 3B) and significantly reduced progeny virus titers (Figure 3C).

Removal of the EGFR from the cell-surface can also be achieved by prolonged stimulation with EGF, causing ligand induced internalization and degradation [17], [18]. To blunt IAV-accessible EGFR from cell surfaces, cells were pre-treated with EGF 90 min before infection. EGF-induced EGFR degradation was monitored in Western-blot analysis (Figure 3D, E). As expected, virion-associated M1 protein levels were strongly reduced in cells where the EGFR was degraded (Figure 3D). This is also reflected by significantly decreased progeny virus titers upon pre-treatment with EGF (Figure 3E). To rule out that modulation of EGFR expression affects the basal EGFR activity and therefore EGFR signaling, phosphorylation of EGFR and phosphorylation of an EGFR regulated down-stream target, ERK2 was monitored in a control experiment (Figure S2). Neither EGFR knock-down nor over-expression of EGFR could induce EGFR or ERK2 phosphorylation (Figure S2). Thus, IAV internalization can be modulated in both directions either by EGFR over-expression or by EGFR knock-down or blockade. Furthermore, the down-regulation of the EGFR expression level did not change the uptake of transferrin, an RTK independent ligand (Figure S1, lower panel), pointing to the specificity of the RTK-regulated endocytosis.

**Figure 4:**
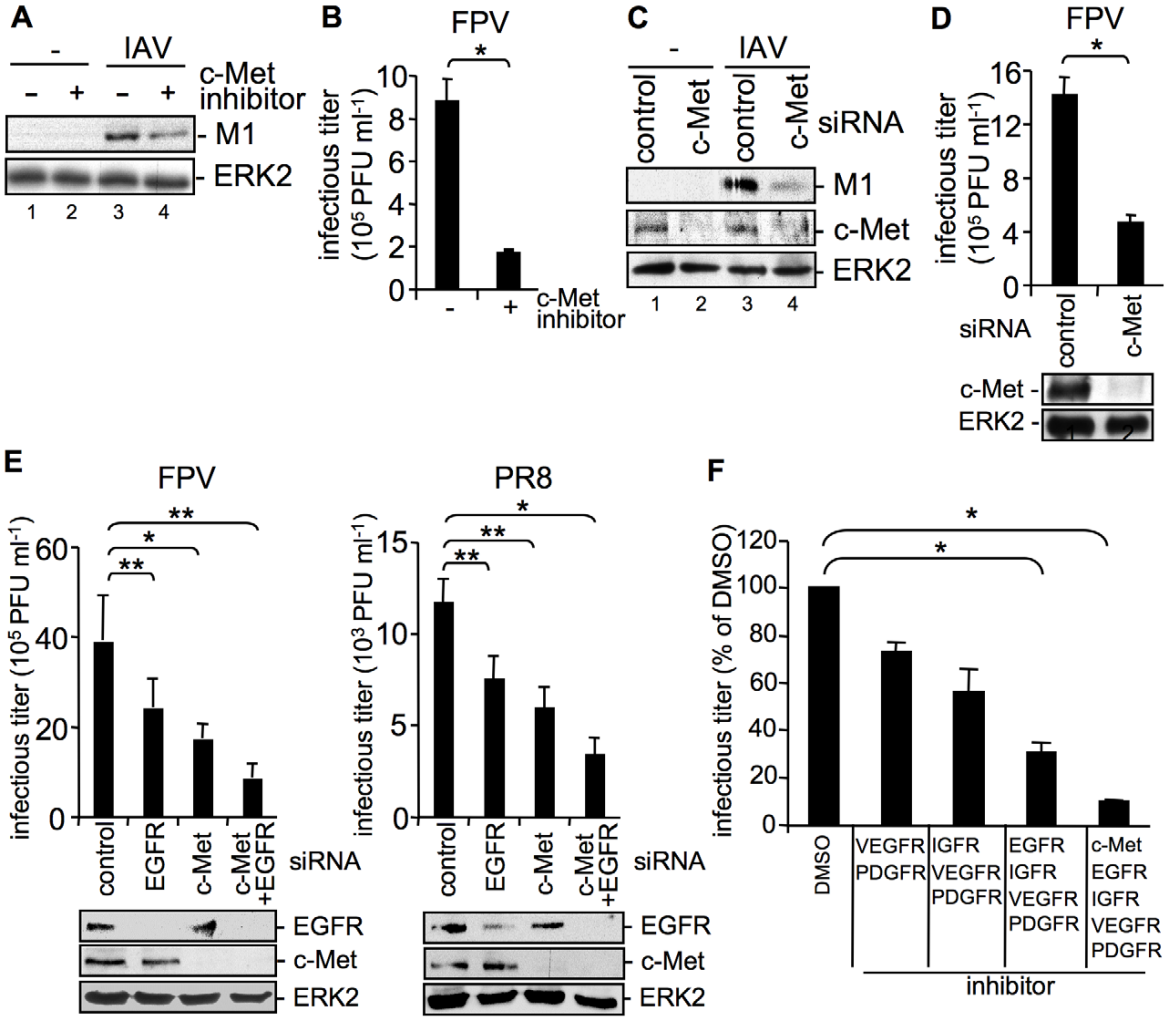
The RTK c-Met is involved in IAV internalization. (A, B) A549 cells were incubated with a c-Met kinase inhibitor (50 µM) at 37°C over night or (C, D) were transfected with control or c-Met specific siRNA 48 h prior to infection. Internalized influenza virus particles were visualized as described in Figure 1B upon infection with the influenza virus strain PR8 (A) (MOI = 8) or (C) (MOI = 4). Progeny virus titers were determined 8 h upon infection with the influenza A virus strain FPV (B, D) (MOI = 4). Data represent mean values of three independent experiments ±SD. Statistical significance was assessed by student's t-test (*) p<0.05, c-Met knock-down was verified by WB before infection (D, lower panel). (E) A549 cells were transfected either with a control siRNA or with specific siRNAs against c-Met and EGFR, as indicated 48 h prior to infection. Progeny virus titers were determined 8 h upon infection with FPV or PR8 (MOI = 4). Data represent mean values of three independent experiments ±SD. Statistical significance was assessed by student's t-test (*) p<0.05, (**) p<0.005. EGFR and c-Met knock-down was verified by WB before infection (E, lower panels). (F) A549 cells were treated with an inhibitor mix containing the indicated RTK inhibitors (each 10 µM, see experimental procedures) or the solvent control (DMSO) 16 hours prior to infection at 37°C. Subsequently cells were infected with FPV (MOI = 4) for 8 h and progeny virus titers were determined. Data represent mean values of three independent experiments ±SD. Statistical significance was assessed by student's t-test (*) p<0.05.

We now addressed the question, whether the effects on IAV entry were exclusively regulated by EGFR or whether other RTKs exhibit similar functions on IAV internalization as suggested by the previous experiments (Figure 1). Indeed, we were able to identify the cellular receptor for hepatocyte growth factor, c-Met as another RTK family member, which is able to interfere with IAV internalization. SiRNA-mediated knock-down of c-Met and inhibition of kinase activity resulted in impaired IAV uptake (Figure 4A, C). The decrease of M1 levels as well as the reduction of progeny virus titers (Figure 4B, D) were similar to that observed upon EGFR inhibition or knock-down, respectively (Figure 3B, C). Moreover, additional experiments using the RTK inhibitor mix indicated that the extent of reduction in virus titers depends on the number of RTKs that were simultaneously affected by specific inhibitors (Figure 4F) or siRNAs prior to infection (Figure 4E). These data indicate that RTK-mediated entry of IAV is not restricted to specific binding and activation of a particular receptor kinase, such as the EGFR but involves a more general mode of activation of several RTKs including the c-Met receptor. We now investigated this mechanism further using the EGFR as a model.

### Attachment of IAV to cells results in clustering of plasma-membrane lipids

**Figure 5:**
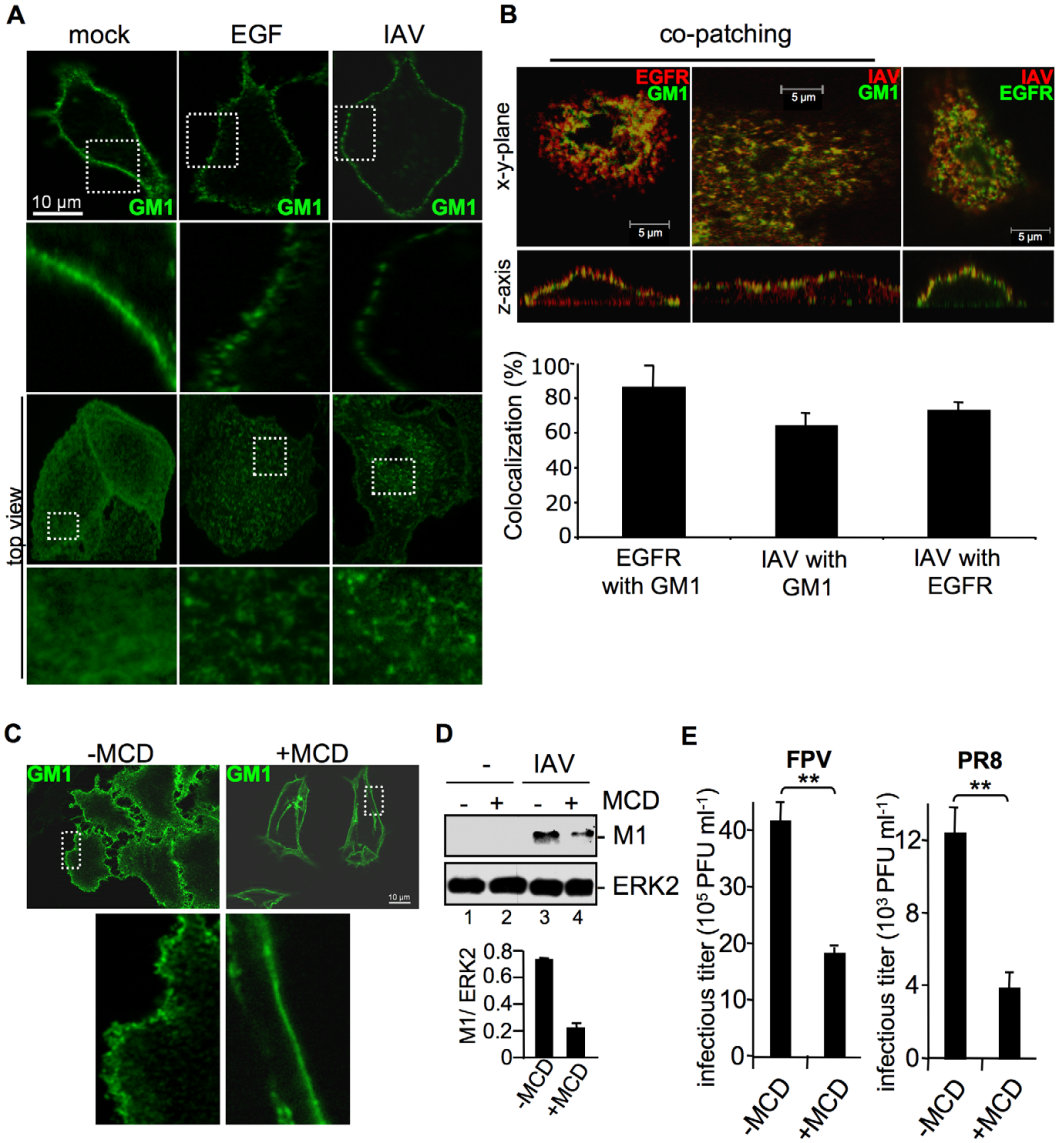
Attachment of IAV clusters plasma-membrane lipids. (A, B) A549 cells were infected with FPV (MOI = 100), stimulated with EGF (100 ng ml^−1^) or left untreated for 1 h at 4°C. Subsequently cells were incubated with the FITC-conjugated Choleratoxin beta subunit (CtxB) (30 µg ml^−1^) for 1 h at 4°C to visualize ganglioside M1 (GM1). (B) FPV was visualized by detection of HA via an HA-specific rabbit antiserum and an Alexa 594-conjugated chicken anti-rabbit IgG (right panel) or via an H7-HA-specific mouse antiserum and a Texas-Red conjugated goat anti-mouse IgG (middle panel). EGFR was detected by an EGFR-specific mouse antiserum followed by a Texas-Red conjugated goat anti-mouse IgG (left panel) or a Alexa 488-conjugated chicken anti-mouse IgG (right panel). For co-patching analysis CtxB was cross-linked by a rabbit antiserum against CtxB. Cells were examined by confocal laser scanning-microscopy. The colocalization was quantified as described in the experimental procedure section. (C, D, E) A549 cells were incubated with methyl-β-cyclodextrin (MCD) (40 µg ml^−1^) for 1 h at 37°C and subsequently washed with PBS to withdraw MCD. (C) Cells were stained for GM1 with FITC-conjugated CtxB for 1 h at 4°C and subsequently incubated with rabbit antiserum against CtxB. (D) Cells were infected with PR8 (MOI = 4) for 1 h at 37°C; an acidic wash (PBS-HCl; pH 1.3, 4°C) was performed. In WB analysis M1 and ERK2 were detected. Relative M1 densities are expressed as mean values ±SD of at least three independent experiments. (E) Cells were infected with FPV or PR8 (MOI = 4); upon the attachment period an acidic wash (PBS-HCl, pH 5.5) was performed. Progeny virus titers were determined by plaque assays. Data represent mean values of three independent experiments ±SD. Statistical significance was assessed by student's t-test (*) p<0.05, (**) p<0.005.

Since EGFR is localized in lipid raft domains [19] and lipid rafts may function as signaling platforms [20], we examined raft organisation in the context of EGF stimulation or viral attachment. The main components of lipid rafts are cholesterol and sphingolipids [21] and these structures are enriched in glycosyl-phosphatidylinositol-linked proteins, signaling molecules as well as the ganglioside GM1. GM1 serves as lipid raft marker. Upon stimulation of EGFR GM1 enriched microdomains undergo endocytosis via clathrin-dependent and clathrin-independent mechanisms [22], [23]. To evaluate whether lipid rafts are clustered upon IAV attachment, we compared GM1 localization upon EGF stimulation or IAV infection of A549 cells. GM1, which is present in the cellular membrane, was labelled using FITC-conjugated Choleratoxin beta subunit (CtxB). In untreated cells GM1 was detected in an even distribution in the cell membrane (Figure 5A, left panel). In EGF treated cells GM1 was reorganized to form distinct patches at the membrane, indicating lipid-cluster formation (Figure 5A, middle panel). Interestingly, the overall staining pattern of GM1 in IAV infected cells (Figure 5A, right panel) closely resembled that observed upon EGF treatment (Figure 5A, middle panel).

To determine whether EGFR is present in lipid rafts upon IAV infection and whether attaching IAV particles are localized to lipid rafts, co-patching experiments were performed. A549 cells were infected or left untreated. Subsequently GM1 was visualized with FITC-conjugated CtxB. Co-patching was performed by crosslinking of CtxB through a rabbit antiserum either in combination to an EGFR-specific or an H7-HA-specific mouse antiserum. The EGFR could be clearly co-patched with GM1, as evidenced in a more than 70% overlap of staining that indicates colocalization (Figure 5B, left panel). This is in the same range as detected for IAV particles (Figure 5B, middle panel). Both, viruses and EGFR were also found to be located in close proximity at the cell surface, here an overlapping staining of more than 80% was observed (Figure 5B, right panel). Thus, the EGFR and attaching IAV particles are localized to lipid raft domains that are reorganized upon infection.

To confirm the requirement of lipid rafts for efficient IAV entry, we depleted cholesterol from the lipid rafts by using methyl-β-cyclodextrin (MCD). First, the efficacy of cholesterol depletion was investigated. Lipid rafts were visualized by detecting GM1 with FITC-conjugated CtxB and raft microdomains were cross-linked using a polyclonal rabbit antiserum against CtxB. After patching of lipid rafts, GM1 was visible in distinct areas at the cell surface (Figure 5C, upper left panel), whereas in MCD treated cells the lipid raft clusters were resolved and GM1 was found to be evenly distributed over the membrane (Figure 5C, upper right panel). To assess a functional link between lipid rafts and IAV internalization, raft microdomains were disrupted by MCD treatment prior to infection. Reduced M1 levels in MCD treated cells indeed indicated impaired virus internalization in the absence of properly formed lipid raft clusters (Figure 5D, lane 4). Impaired virus uptake provoked by cholesterol depletion prior to infection led to significantly reduced virus titers (Figure 5E).

### The EGFR undergoes endocytosis in response to IAV attachment

**Figure 6:**
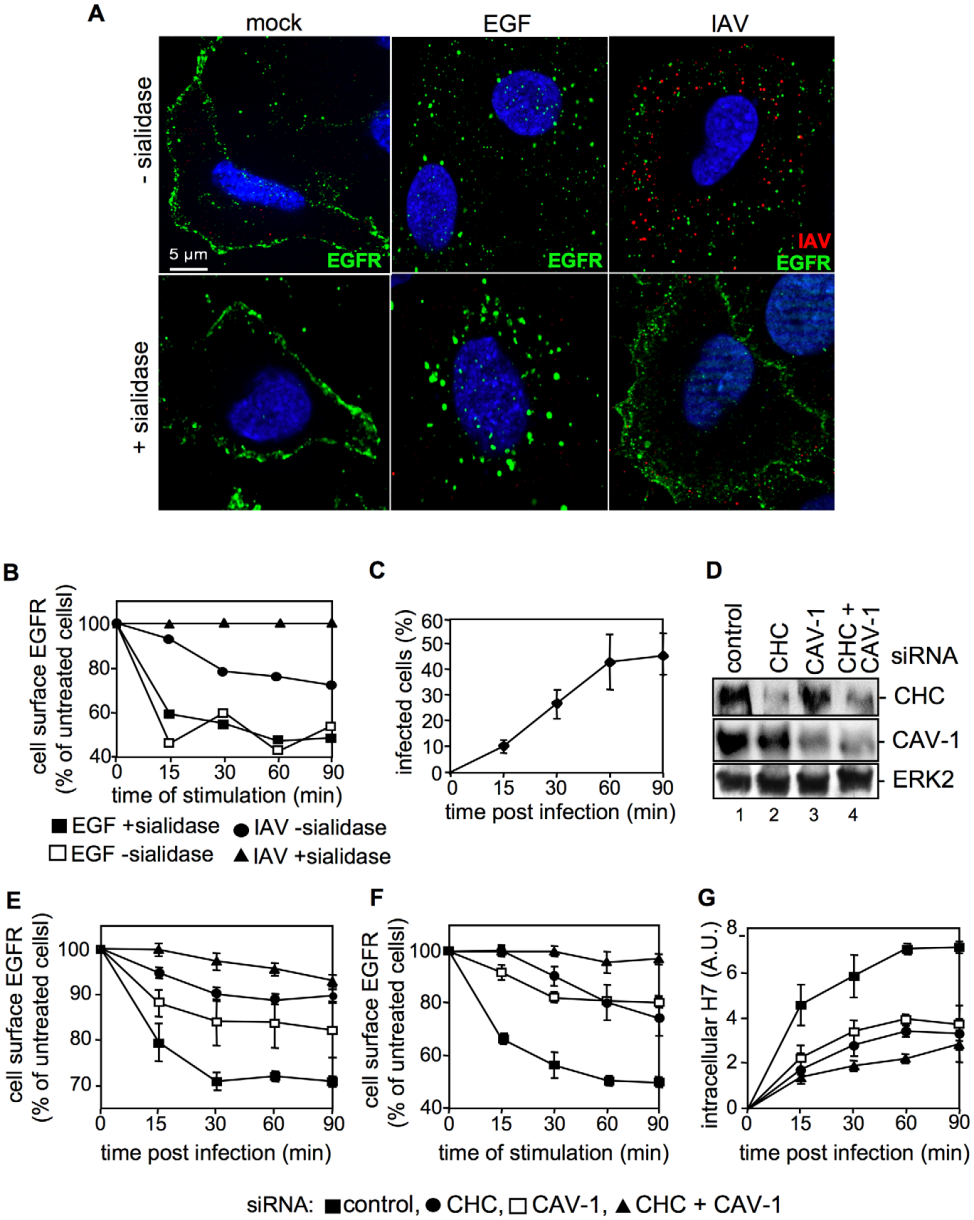
IAV attachment induces EGFR endocytosis. (A) Upon treatment with sialidase (± sialidase; 0.01 units ml^−1^, 3 h, 37°C), A549 cells were infected with FPV (MOI = 100) or were stimulated with EGF, each for 1 h at 4°C and 30 min at 37°C. An EGFR-specific mouse antiserum and Alexa 488-conjugated chicken anti-mouse IgG as well as a HA-specific rabbit antiserum and an Alexa 594-conjugated chicken anti-rabbit IgG were employed. Cells were examined by IF microscopy. (B, C) A549 cells were infected with FPV (MOI = 100) or (B) incubated with EGF (30 ng ml^−1^) for 1 h at 4°C upon treatment with sialidase (± sialidase; 0.01 units ml^−1^, 3 h, 37°C). After a temperature shift cells were further kept at 37°C for the indicated times. (B) Surface resident EGFR was detected by FACS analysis. Fluorescence of uninfected/-stimulated cells was arbitrarily set as 100%. (C) After an acidic wash (PBS-HCl, pH 1.3), cells were permeabilized with saponin (0.2% w/v). Infected cells were assessed by detection of viral HA in FACS analysis. (D, E, F, G) A549 cells were transfected with a control or specific siRNAs targeting caveolin-1 (CAV-1) or clathrin heavy chain (CHC). (D) 48 h post transfection, knock-down was verified by WB using the indicated antibodies. (E, F) upon knock-down cells were (E) infected with FPV or (F) incubated with EGF as described in (B), without sialidase treatment. Cell surface EGFR was detected as mentioned in (B). (G) FPV internalization upon knock-down of the indicated proteins was analysed as described in (C).

So far our results indicate the presence of EGFR and IAV particles in close proximity within lipid rafts upon viral attachment. We now examined whether the EGFR is also activated upon virus binding to the cell surface. If this would be the case one would expect that the EGFR undergoes endocytosis upon IAV attachment, similar to EGF stimulation. Cells were either left untreated (Figure 6A, left panel), stimulated with EGF (Figure 6A, middle panel) or infected with IAV (Figure 6A, right panel). In an additional set, sialic acids were removed by sialidase treatment to prevent virus attachment (Figure 6A, lower panel). In IF-microscopy we observed EGFR located at the plasma membrane in untreated cells (Figure 6A, left panel). As expected the receptor was internalized upon EGF stimulation (Figure 6A, upper middle panel), a process that was independent from sialidase treatment (Figure 6A, lower middle panel). Upon IAV infection of cells both, virus particles and the EGFR were internalized (Figure 6A, upper right panel). While at the time of virus entry EGFR and IAV are colocalized (Figure 5B) IAV and EGFR colocalization is not detectable anymore at 30 min p.i.. This observation presumably point to a distinct sorting of IAV and EGFR. In sialidase treated cells neither virus attachment nor EGFR internalization was visible (Figure 6A, lower right panel), indicating that IAV attachment promotes EGFR internalization in a sialic acid dependent manner. These results could be confirmed by FACS analysis of cell surface associated EGFR. Sialidase treated or untreated cells were stimulated either with EGF or infected with IAV. Again, the internalization of EGFR upon EGF stimulation was not sensitive to sialidase treatment, as evidenced by reduced amounts of surface located EGFR. IAV infection also triggered EGFR endocytosis albeit to a lower extend and in a sialidase dependent manner (Figure 6B). According to the kinetic of EGFR internalization, EGF treatment leads to a strong decrease in the first 15 min turning to a plateau later (Figure 6B). In contrast, IAV attachment reduced the number of EGFR at the cellular surface in a linear fashion (Figure 6B), which matches the linear increase of internalization detected for IAV (Figure 6C). These results point to a similar internalization kinetic for IAV and EGFR during infection. Further analysis of the internalization pathways utilized by IAV and EGFR in infected cells revealed that both equally depend on clathrin and caveolin-1 expression. SiRNA-mediated knock-down of either clathrin heavy chain (CHC) or caveolin-1 (CAV-1) (verified by Western blot, Figure 6D) in IAV infected cells resulted in a decrease of cell-surface EGFR of about 10% or 15%, respectively, at 30 min p.i. (Figure 6E). Upon simultaneous knock-down of CHC and CAV-1 the amount of EGFR located at the cellular surface, was only reduced about 3% after infection (Figure 6E) indicating that internalisation is greatly dependent on these two factors. In another experimental setting it could be shown that the dependency on CHC and CAV-1 is similar for EGF stimulated and virus-induced EGFR internalisation (Figure 6F). The measurement of internalized IAV particles verified the strong dependency of IAV on CHC and CAV-1 during IAV uptake processes (Figure 6G). Upon knock-down of CAV-1 or CHC expression IAV internalization was reduced and concurrent knock-down of CAV-1 and CHC inhibited IAV uptake, further (Figure 6G).

### IAV attachment to A549 cells induces EGFR kinase activity

**Figure 7:**
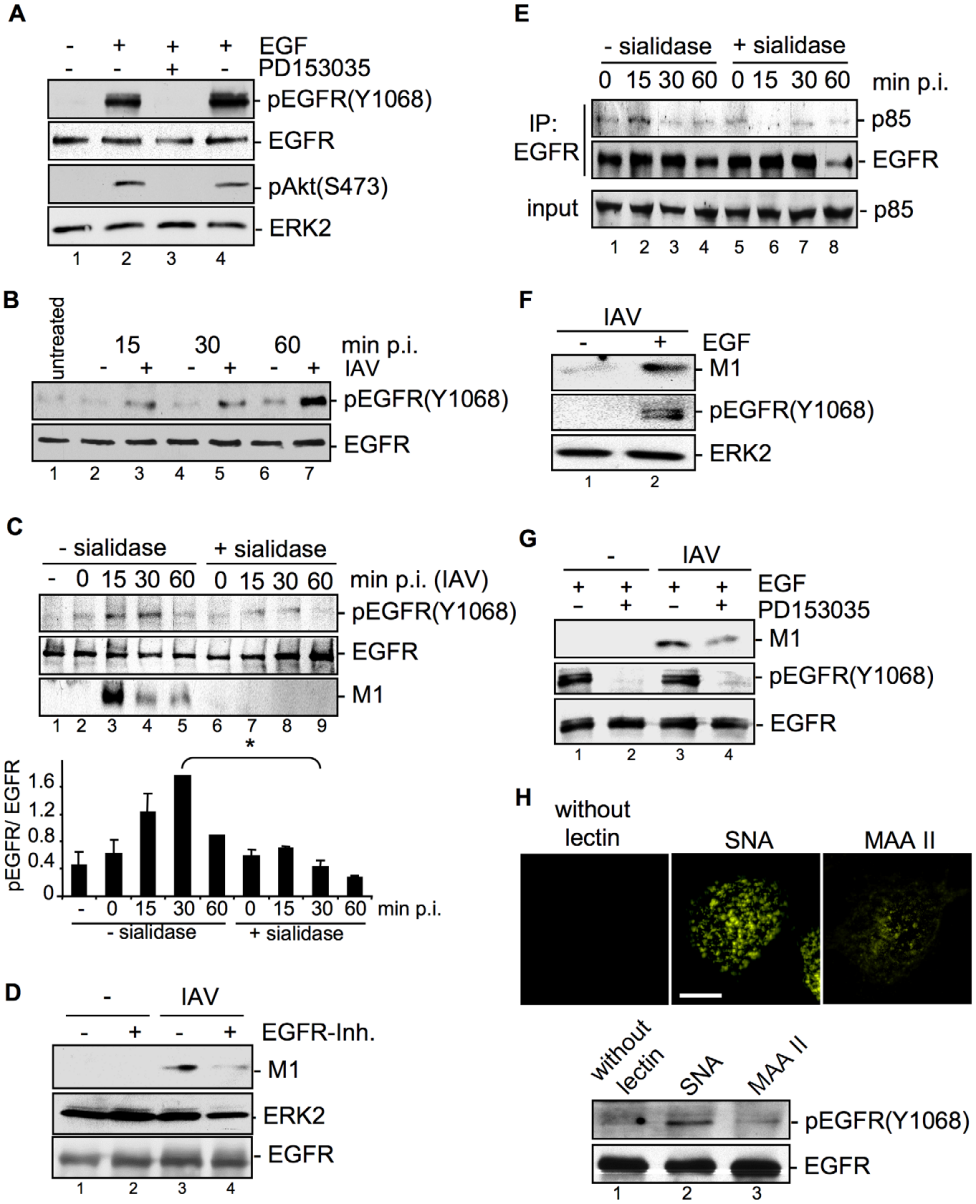
EGFR kinase activity is induced upon viral attachment and is required for efficient IAV internalization. (A) A549 cells were incubated with PD153035 (2.8 µM) or DMSO for 1 h at 37°C and were subsequently treated for 10 min with EGF (30 ng ml^−1^). PI3K dependent Akt phosphorlyation (pAktS473) and phosphorylated EGFR (pEGFRY1068) were detected by WB. (B) A549 cells were mock infected [−] or infected with FPV (MOI = 100) [+] for the indicated times at 37°C. In (C and E) cells were treated with sialidase (± sialidase; 0.01 units ml^−1^, 3 h, 37°C), and inoculated with PR8 (MOI = 100) for 1 h at 4°C. Subsequently cells were incubated at 37°C for the indicated times. (C, lower panel) The amount of pEGFR(Y1068) was quantified. Relative pEGFR(Y1068) densities are expressed as mean ±SD of three independent experiments. (D, G) A549 cells were infected with PR8 (MOI = 4) for 30 min upon pre-treatment with (D) an EGFR inhibitor (10 µM), or (G) PD153035 (2.8 µM) or DMSO for 1 h at 37°C. (E) EGFR was immuno-precipitated. Co-immunoprecipitated p85 and the input-control was analysed in WB. (F) Cells were stimulated with EGF (30 ng ml^−1^) 10 min prior to infection and infected with PR8 (MOI = 4) for 30 min at 37°C; an acidic wash (PBS; pH 1.3, 4°C) was performed. WBs were probed with the indicated antibodies. (H) Biotinylated *Sambucus nigra* agglutinin (SNA) or *Maackia amurensis* agglutinin II (MAAII) (1 µg ml^−1^) were incubated for 60 min at 4°C with A549 cells. Subsequently cells were either incubated at 37°C for 30 min and lysed for detection of pEGFR(Y1068) levels by WB or incubated with Cy3-conjugated streptavidin to detect lectins for fluorescence microscopy (scale bar 10 µm). See also Figure S3.

Together, the data so far suggest that IAV attachment leads to clustering of lipid rafts and stimulates internalization of EGFR. Considering the requirement of EGFR kinase activity for EGF-induced EGFR endocytosis [24], [25] these results imply that virus attachment should induce EGFR kinase activation. Since previous studies revealed the requirement of PI3K activity for efficient IAV uptake [6], we focussed on tyrosine 1068 (Tyr1068) of EGFR, a known EGF inducible autophosphorylation site that serves as a docking site for the protein Gab1 leading to PI3K recruitment [26]. Tyr1068 readily gets phosphorylated in response to EGF stimulation of A549 cells, concomitant with phosphorylation of the PI3K effector Akt at Ser473, a site that is targeted in a strictly PI3K dependent manner (Figure 7A, lane 2 and lane 4). Both, phosphorylation of the EGFR and Akt could be blocked by PD153035, a specific EGFR kinase inhibitor (Figure 7A, lane 3). This indicates that PI3K activation via EGF in A549 cells requires EGFR kinase activity and autophosphorylation at Tyr1068.

The next set of experiments should elucidate whether EGFR activity is induced upon IAV infection and whether EGFR activity is required for virus uptake. Western-blot analysis revealed that EGFR was time-dependently phosphorylated upon IAV infection (Figure 7B). Virus-induced EGFR phosphorylation occurred concomitant with accumulation of virus-associated M1 (Figure 7C, lane 3 to 5) and was dependent on viral binding to sialic acids as evidenced by disappearance of the phospho-signal upon pre-treatment with sialidase (Figure 7C, lane 7 to 9). Inhibition of the EGFR kinase by an EGFR specific inhibitor resulted in reduced M1 abundance indicating the requirement of kinase activity for virus internalization (Figure 7D). This was also visible by reduced infection upon inhibiton of the EGFR kinase prior to infection applying either the PD-inhibitor or Lapatinib, an additional EGFR inhibitor (data not shown). In line with these data recruitment of the regulatory subunit p85 of PI3K to the EGFR was observed in response to viral attachment (Figure 7E, lane 2). In addition, enhanced EGFR activity that was achieved by pulse stimulation with EGF for 10 min resulted in enhanced virus uptake (Figure 7F, lane 2). This effect was reversible by the EGFR kinase specific inhibitor PD153035 (Figure 7G, lane 4). Taken together, our results clearly indicate that active EGFR promotes the IAV entry processes. The data further suggest that EGFR gets activated by formation of raft signaling domains due to multivalent binding of the viral HAs to sialic acids at the cell surface. Concomitant, we could show that IAV induced EGFR-signaling, which regulates PI3K activation, only occurs in cells with intact raft domains (Figure S3, lane 2). In accordance with previous studies [27], [28] the aberrant phosphorylation of EGFR by MCD was not transmitted into the downstream signaling leading to the activation of PI3K (Figure S3, lane 4 and 5). Thus, EGFR phosphorylation is not sufficient for full induction of downstream signaling, but rather depends on functional raft domains. To analyse whether such a clustered binding of sialic acids is sufficient to activate the EGFR kinase, we made use of lectins. These compounds bind sialic acids in a multivalent fashion and cause hemagglutination similar to IAV [29]. The lectin *Sambucus nigra* agglutinin (SNA) specifically recognizes Siaα2-6Gal, while *Maackia amurensis* agglutinin II (MAAII) is specific for Siaα2-3Gal linkage [30], [31]. SNA staining revealed that Siaα2-6Gal is present on A549 while Siaα2-3Gal was detected with MAAII to lower amounts (Figure 7H, upper panel). Accordingly binding of SNA resulted in an activation of EGFR kinase (Figure 7H, lower panel, lane 2), whereas MAAII that is not bound failed to induce EGFR kinase activity (Figure 7H, lower panel, lane 3).

## Discussion

Influenza viruses are cell obligate parasites and thus, are strongly dependent on the cellular machinery of their host. In case of IAV the initial steps of infection comprise the binding to sialic acids at the cellular membrane and subsequent internalization of the virus particles. The requirement of active cellular signaling processes for internalization, such as activation of PI3K [6], suggested that IAV penetration of the cellular membrane is not a constitutive process, but is supported by immediate signaling events induced by viral binding to the cell surface.

Requirement of post-attachment factors to achieve efficient endocytosis of IAV has already been discussed previously [3]. In the present study we could identify the EGFR and the c-Met receptor as candidate RTKs that are activated upon attachment and promote IAV internalization.

RTKs came into focus as prime candidates for signaling receptors involved in IAV uptake, since kinases such as PI3K or ERK1/2, which are known to be activated directly upon virus binding [5], [6] are targeted by these receptors. To unravel the mechanism of RTK function during IAV uptake, we exemplarily studied the EGFR as an abundantly expressed signaling-receptor model that is able to induce PI3K activity. We could not only demonstrate that the EGFR kinase is activated by IAV attachment but also showed that the active kinase is involved in promotion of initial IAV uptake into host cells. The same holds true for the c-Met receptor tyrosine kinase. These results indicated that activation of RTKs such as EGFR and c-Met is a crucial step for efficient IAV entry, rather than a by-standing effect. The findings further imply that activation is not mediated by viral engagement of a particular receptor kinase but is a more general phenomenon that affects several RTKs. This is additionally supported by results of a recent siRNA screening study, which identified the fibroblast growth factor receptors FGFR 1, 2 and 4 as RTKs involved in very early steps of viral infection [32].

**Figure 8:**
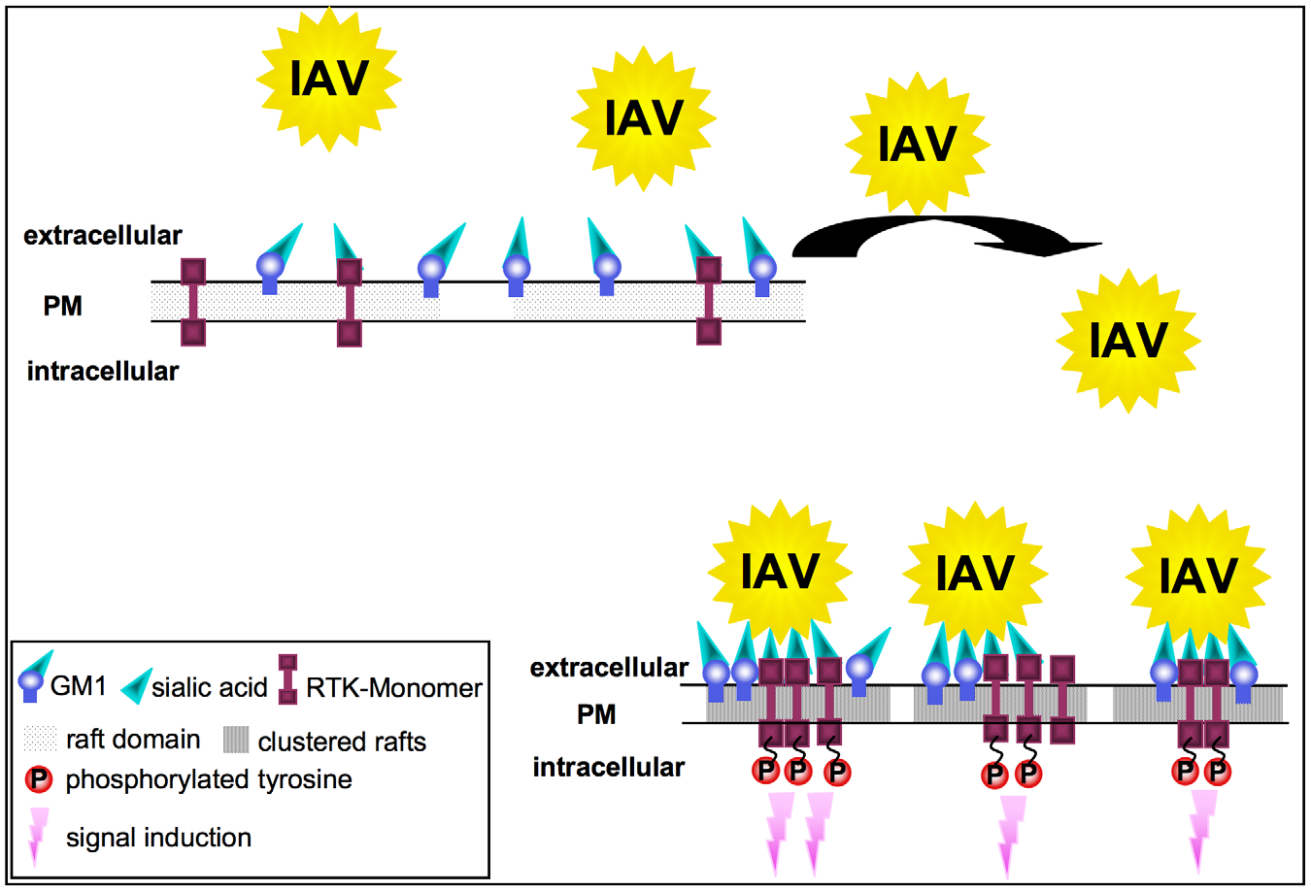
Model for the RTK-mediated internalization of IAV. In unstimulated cells, RTKs are localized in GM1 positive lipid rafts at the plasma membrane. Multivalent binding of viral HA to sialic acids at RTKs or GM1 leads to the aggregation of rafts, resulting in the concentration and clustering of RTK. Intrinsic kinase activity of RTKs is triggered in response to clustering. This leads to signal induction and presumably to the onset of endocytosis of the RTKs, which facilitates IAV internalization.

EGF stimulation induces aggregation of lipid rafts, serving as signaling platforms to initiate signal transduction and EGFR internalization [33]. Our data provide indirect experimental evidence for similarities between EGF and IAV induced lipid cluster formation (Figure 5A). Therefore it seems likely that IAV leads to a concentration of signaling modules within clustered lipid rafts and thereby inducing signal transduction, as shown for other ligands [34]. Such a mechanism was also suggested for EGFR activation [35]. Even if previous results by Whittaker and colleagues [36], [37] demonstrated that cholesterol depletion from the virus envelope but not from cellular membrane resulted in reduced virus infection, in our hands cholesterol depletion of A549 cells impaired efficient IAV internalization (Figure 5D). These divergent results are probably due to different experimental settings, the usage of divers cell-lines and various influenza virus strains. The most essential difference is referred to the distinct treatment of cells with MCD. While Whittaker and colleagues added MCD during both, the virus adsorption and incubation periods [36], in our experimental settings cells were pre-incubated with MCD for 60 min before infection and MCD was removed afterwards. Based on our results we hypothesize that IAV induce raft cluster formation by the multivalent binding of viral HA to sialic acids. The clustered rafts serve as signaling platforms to facilitate virus internalization. Sialic acid-modified GM1, analysed in the present study (Figure 5) is in particular a suitable interaction partner for viral HA and probably triggers arrangement of signaling platforms (Figure 8). However, we observed only partial colocalization of IAV and GM1 at the cell surface. These results point to multiple internalization mechanisms used by IAV, even independent from raft microdomains.

Formation of clusters upon IAV attachment probably brings raft resident signaling molecules such as RTKs in close proximity, which are subsequently activated (Figure 8). A remaining challenge concerned the mechanism of EGFR mediated IAV uptake into the host cells. Based on our results we considered a facilitated IAV entry accompanied by down-regulation of signaling receptors that presumably would promote co-endocytosis of IAV into the host cell. Indirect evidence was provided by the observation of an attachment dependent internalization of EGFR and IAV triggered down-regulation of EGFR by onset of endocytotic processes (Figure 6A, B). Several phosphorylated tyrosine residues at the trans-phosphorylation site of the EGFR were described as binding sites for factors involved in the ligand-induced removal of the EGFR from the cell surface. Among these factors are c-Cbl [38], [39] AP2 and Epsin15 [40], [41] and PI3K [42]. Furthermore, the data indicate an equal contribution of clathrin- and caveolin dependent endocytosis during IAV uptake and IAV induced EGFR internalization (Figure 6E, G). Based on these results we concluded that clathrin- and caveolin-1 are additional regulatory factors, which mediate IAV internalization in cooperation with RTKs. In this study we identified an IAV induced generation of a binding site for PI3K at the EGFR. Upon IAV infection Tyr1068 at the EGFR was phosphorylated, which generates a putative binding site for the regulatory subunit of PI3K, p85 [43]. In fact, we observed a recruitment of p85 to the EGFR in response to IAV attachment. One remaining question would be, whether there is an involvement of additional effector proteins beyond PI3K, e.g. the E3 ubiquitin ligase c-Cbl, which is required for progression of EGFR into clathrin coated pits upon ligand binding [44]. It is likely that a binding site for c-Cbl at the EGFR will be generated upon IAV attachment and triggers viral particle sorting into endocytotic pathways upon stimulation. This hypothesis is supported by recognition of an ubiquitin-vacuolar protein sorting system for IAV [13] and recruitment of c-Cbl to the plasma membrane upon EGFR activation [45]. Additional analyses revealed that also other sites of EGFR-family members such as Tyr1112 and Ser1113 are phosphorylated upon IAV attachment similar to EGF stimulation (Figure S4).

In summary, our data provide first evidence for a signaling regulated uptake of IAV involving activation of RTKs. Multivalent binding of the HA to sialic acids at the cell surface appear to trigger raft formation, which brings receptor monomers in close proximity and induce a certain level of RTK activation. This hypothesis was supported by the finding that SNA binding mediates EGFR kinase activation since it is described that SNAs harbour at least two binding sites for sialic acids [30], and therefore share similarities with the multivalent nature of IAV. RTK activity in turn leads to activation of intracellular signaling molecules, such as PI3K that promotes uptake. Because EGFR and c-Met are only two receptors among many other signaling receptors that may be activated and may affect the entry of IAV, the titer reduction in EGFR or c-Met knock-down experiments was not in the range of log-steps. However, the magnitude of the effects obtained by functional interference with one or two RTKs are in line with the mechanism proposed that several RTKs are involved during virus internalisation. This model is fully supported experimentally by a simultaneous knock down of c-Met and EGFR (Figure 4E) or a stepwise inhibition that resulted in additive effects on viral replication (Figure 4F). However, at the same time these experiments demonstrate a striking limitation in terms of co-affecting RTKs with regard to a potential antiviral approach. Just a limited number of RTKs can be manipulated by chemical inhibitors or siRNAs at the same time, without interfering with the viability of cells and thus titer reductions in the range of several log steps cannot be expected.

In summary, the wide variety of different experiments shown here, including Western blot analysis, virus internalisation assays on a single cell level and infection experiments with various MOIs consistently demonstrate that IAV internalization is promoted by RTKs, such as the EGFR or c-Met receptor.

## Materials and Methods

### Cells, viruses and viral infections

Madin Darby Canine Kidney (MDCK) cells were grown in MEM, the human lung epithelial cell line A549 and the mouse embryonic fibroblasts cell line NIH3T3 were grown in DMEM. Her14 are NIH3T3 cells stably expressing the human wild-type EGFR [16]. The cell culture media were supplemented with 10% heat-inactivated fetal bovine serum (FBS) and antibiotics. Cells were cultured at 37°C and 5% CO_2_. Avian influenza virus A/FPV/Bratislava/79 (H7N7) (FPV) and human influenza virus A/Puerto Rico/8/34 (H1N1) (PR8) were originally provided by the Institute of Medical Virology in Giessen, Germany. Virus strains were propagated on MDCK cells supplemented with MEM without FBS but 0.2% bovine serum albumine and antibiotics; supernatants were cleared by centrifugation at 6.000 g for 10 min at 4°C, loaded onto a sucrose cushion (20% (w/v)) and centrifuged at 20.000 g for 3 h at 4°C. IAV was adsorbed as described in the figure legend. Afterwards, cells were either washed with PBS or acidic PBS, dependent on the experimental procedure that is indicated in the figure legend. A harsh acidic wash (PBS-HCl, pH 1.3 at 4°C) was included to prevent detection of attached, but not internalized virus particles in the entry assays. For determination of progeny virus titers, cells were inoculated with virus for 30 min at 37°C to allow virus attachment and internalisation. Afterwards a mild acidic wash (PBS-HCl, pH 5.5 at 4°C) was performed to exclude the delayed uptake of attached, but not internalized virus particles. Subsequently cells were incubated at 37°C for further 8 hours or the times indicated in the figure legend. Afterwards the supernatant was taken and the number of infectious virus particles was determined by standard plaque assay, as described previously [15]. A pairwise, two-tailed student's t-test was performed for statistical comparison between the samples.

### Plasmids, reagents and inhibitors

The sequence encoding human EGFR, fused to cyan-fluorescent protein in pECFP-C1 was kindly provided by Dr. N. Zobiack (Institute for Medical Biochemistry, ZMBE, Münster, Germany). EGFR-, c-Met-, caveolin-1-, clathrin heavy chain-specific siRNAs were purchased from Qiagen. Cells were transfected with Lipofectamine 2000 (Life Technologies) according to a protocol described previously [46]. The following reagents were purchased from the suppliers indicated and used as described in the figure legends: human recombinant EGF (R&D systems Minneapolis, USA), sialidase (Roche), DMSO and genistein (Sigma-Aldrich, Germany) a general inhibitor of tyrosine kinases, the EGFR-blocking antibody (clone O.N. 265) (USBiological), and the EGFR inhibitors from Calbiochem (#324674, PD153035). Biotinylated *Sambucus nigra* agglutinin (SNA), biotinylated *Maackia amurensis* agglutinin II (MAAII) and Cy3-conjugated streptavidin were purchased from Linaris (Wertheim, Germany). The RTK inhibitor mix contained the following kinase inhibitors: SU4312 (Santa Cruz Biotechnology) targeting plateled derived growth factor receptor (PDGFR) and vascular endothelial growth factor receptor (VEGFR), Picropodophyllin (Calbiochem) targeting the insulin growth factor receptor (IGFR), SU11274 (Calbiochem) a Met-kinase inhibitor and Gefitinib (Santa Cruz Biotechnology) that represents an EGFR specific kinase inhibitor (each 10 µM).

### Immunoprecipitation and Western-blotting

Immunoprecipitation (IP) and Western-blotting (WB) procedures were performed as described earlier [6]. For EGFR IP a polyclonal anti-EGFR antibody (Santa Cruz Biotechnology) and protein A agarose (Roche) were employed. The viral matrix protein 1 (M1) was visualized with a mouse monoclonal M1 antiserum (Serotec). Endogenous and over-expressed EGFR was detected by an EGFR-specific rabbit antiserum (Cell Signaling Technology). Phosphorylated forms of EGFR, Akt and ERK2 were detected by different phosphospecific antisera: phosphospecific EGFR(Y1068) mouse antiserum (Cell Signaling Technology), phosphospecific Akt(S473) rabbit antiserum (Biosource) or phosphospecific ERK1/2 (pT202/pY204, pT185/pY187) mouse antiserum (Cell Signaling Technology). The regulatory subunit p85 of PI3K, was detected by a mouse monoclonal p85 antiserum (Serotec). In some of the assays, loading controls were performed with pan-ERK2 antiserum (Santa Cruz Biotechnology) or pan-Akt antiserum (Cell Signaling Technology). c-Met was detected by a c-Met specific rabbit antiserum (Santa Cruz Biotechnology). Knock-down of caveolin-1 and clathrin heavy chain was verified by WB using a caveolin-1-specific rabbit antiserum (Santa Cruz Biotechnology) and clathrin heavy chain-specific mouse antiserum (BD Transduction Laboratories). For screening of additional phosphorylation sites in the EGFR the Human EGFR Phosphorylation Antibody Array 1 from RayBio (USA) was employed according to the manufacturers instructions.

### Quantification of Western-blots

Western-blot experiments were quantified using Advanced Image Data Analyser Software (AIDA, Raytest GmbH, Straubenhardt, Germany). The total band densities were measured against the local background. Obtained values of M1 or pEGFR(Y1068) were divided through the values of the ERK2 and EGFR loading control, respectively. All data were expressed as means ±SD of three independent experiments.

### Indirect immunofluorescence microscopy

Indirect immunofluorescence microscopy was performed as described earlier [6]. Top view images were prepared as compacted Z-Stack images of non-permeabilised cells, using Zeiss AxioVision software. For colocalization analysis, cells were examined by confocal laser-scanning microscopy. Quantification and statistical analysis of colocalization events was calculated as described in [47]. We used BioImageXD-free open source software for analysis and visualization of multidimensional biomedical images (http://www.bioimagexd.net). X-y plane and z-axis views of confocal images were prepared using ZEN 2009 LE software from Zeiss, Germany.

### Co-patching of lipid rafts with proteins of interest and colocalization-experiments

FITC-conjugated Choleratoxin beta subunit (CtxB) (Sigma) was used to visualize raft resident ganglioside M1 (GM1) upon infection. Subsequently lipid rafts were cross-linked with a rabbit antiserum against CtxB (Sigma). Samples were incubated for 1 h at 4°C and subsequently for 15 min at 37°C. EGFR and viral HA were visualized by the common immunofluorescence staining protocol. An EGFR specific mouse antiserum (Calbiochem), a (HA)-specific rabbit antiserum and a H7-HA mouse antiserum, respectively (kindly provided by H.D. Klenk, University of Marburg, Germany) were used. Methyl-β-cyclodextrin (MCD) (Sigma, Aldrich) was used to deplete cholesterol as described previously [48].

To visualize other proteins in immunofluorescence microscopy an antibody against the early endosomal antigen 1 (EEA1) (Acris) and TexasRed labelled transferrin (30 µg ml^−1^) (Invitrogen) were used.

### Flow cytometry analysis (FACS)

A549 cells were infected with IAV strain FPV or stimulated with EGF, harvested and prepared for flow cytometry analysis with FACSCalibur (Becton Dickinson). Cells were washed with cold PBS, centrifuged for 2 min at 500 g and subsequently incubated with 1.85% formaldehyde for 20 min at room temperature. For intracellular protein stains, cells were permeabilized with 0.2% w/v saponin (Sigma-Aldrich) for 10 min at room temperature. Cells were treated with 1% fetal calf serum and incubated with an EGFR specific mouse antiserum (Calbiochem) or an H7-HA mouse antiserum (kindly provided by H.D. Klenk, University of Marburg, Germany). After several washing steps, cells were incubated with an Alexa Fluor 488 chicken anti-mouse IgG (H+L). Cells were resuspended in PBS and subjected to FACS analysis.

## Supporting Information

Figure S1 Transferrin uptake occurs independently from genistein treatment or EGFR knock-down upon siRNA transfection. A549 cells were treated with genistein (50 µM) for 60 min prior to infection (upper panel) or were transfected for 48 h with control siRNA and specific siRNA targeting EGFR, respectively (lower panel). Afterwards cells were incubated with TexasRed-labelled transferrin (30 µg/ml) or EGF (30 ng/ml) for 10 min at 37°C. EGFR was detected by an EGFR-specific mouse antiserum and EEA1 was detected by an EEA1 rabbit antiserum followed by a Texas-Red conjugated goat anti-mouse IgG or an Alexa 488-conjugated chicken anti-rabbit IgG, respectively. Cells were examined by confocal laser scanning-microscopy. The colocalization was quantified as described in the experimental procedure section.
(4.39 MB TIF)

Figure S2 Modulation of the overall EGFR number does not alter the basal EGFR-signaling. A549 cells were left untransfected (lane 1, 2) or were transfected with siRNA (lane 3, 4) for 48 h or expression constructs (lane 5, 6) for 24 h hours and subsequently lysed. Lysates of untransfected cells, which were stimulated with EGF (30 ng/ml, 10 min) served as positive control (lane 1). WBs were probed with the indicated antibodies.
(0.36 MB TIF)

Figure S3 Cholesterol depletion inhibits IAV-induced EGFR downstream signaling. A549 cells were left untreated or were treated with sialidase (0.01 units/ml, 3 h, 37°C). After 2 h incubation with sialidase, MCD (40 µg/ml) was added in one untreated and one sialidase treated probe. After incubation for another hour at 37°C, PR8 (MOI = 100) was attached at 4°C for 60 min and subsequently incubated for further 30 min at 37°C, before cell-lysis. WBs were probed with the indicated antibodies.
(0.23 MB TIF)

Figure S4 Screening for additional EGF or virus-induced phosphorylation sites in EGFR family members. A549 cells were either left untreated or incubated with EGF (30 ng/ml) or infected with IAV strain PR8 (MOI = 100) for 15 min at 37°C. Cell lysates were analysed for different phosphosites in different EGFR-family members, using the Human EGFR Phosphorylation Antibody Array 1 from RayBio (see material and methods).
(0.36 MB TIF)
