# Supplementary figures and images for: The Epidermal Growth Factor Receptor (EGFR) Promotes Uptake of Influenza A Viruses (IAV) into Host Cells
Source: PLoS Pathog. 2010 Sep 9;6(9):e1001099. doi: 10.1371/journal.ppat.1001099 (PMC2936548; doi:10.1371/journal.ppat.1001099)

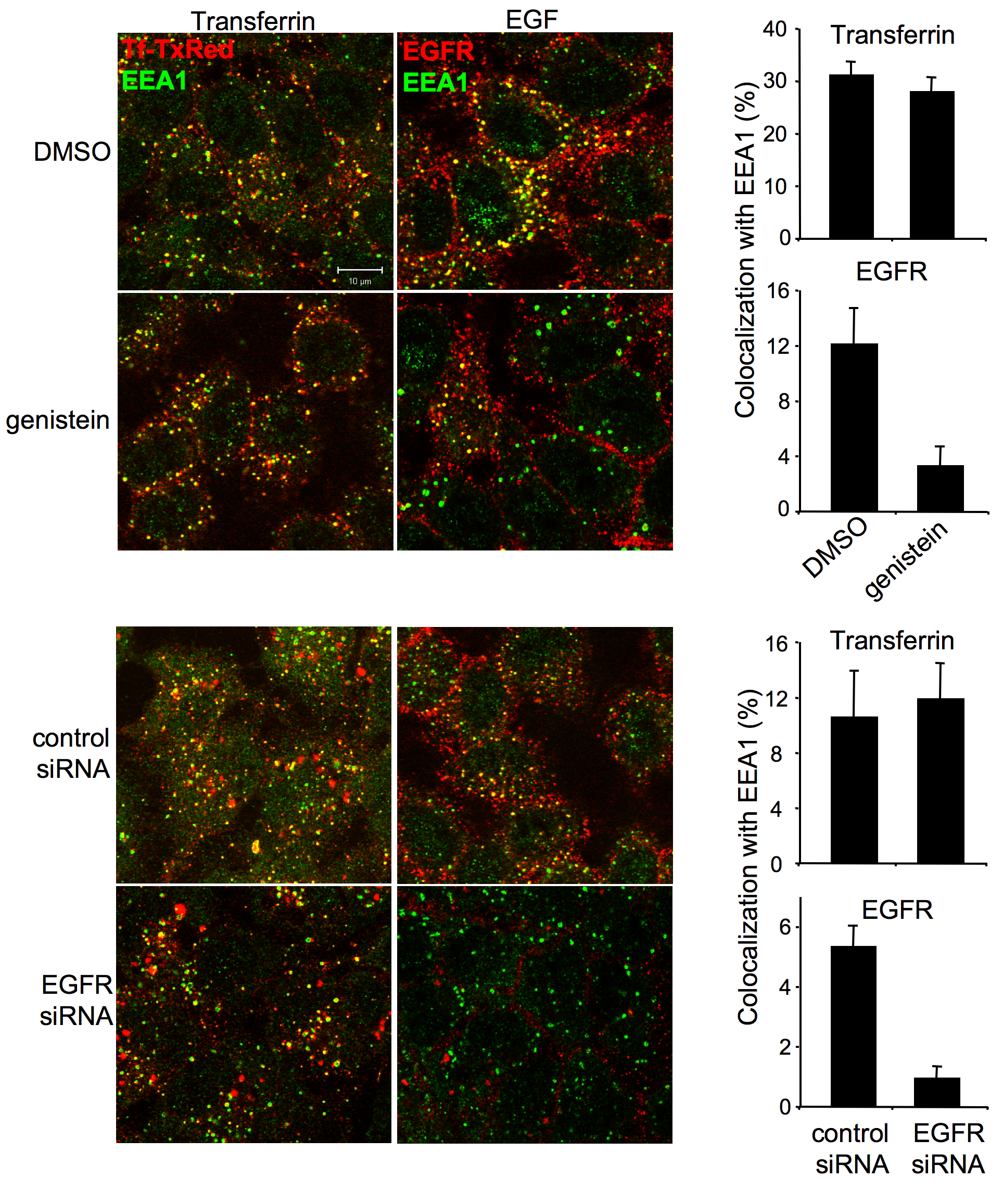

Supplement: Figure S1 — Transferrin uptake occurs independently from genistein treatment or EGFR knock-down upon siRNA transfection. A549 cells were treated with genistein (50 µM) for 60 min prior to infection (upper panel) or were transfected for 48 h with control siRNA and specific siRNA targeting EGFR, respectively (lower panel). Afterwards cells were incubated with TexasRed-labelled transferrin (30 µg/ml) or EGF (30 ng/ml) for 10 min at 37°C. EGFR was detected by an EGFR-specific mouse antiserum and EEA1 was detected by an EEA1 rabbit antiserum followed by a Texas-Red conjugated goat anti-mouse IgG or an Alexa 488-conjugated chicken anti-rabbit IgG, respectively. Cells were examined by confocal laser scanning-microscopy. The colocalization was quantified as described in the experimental procedure section. (4.39 MB TIF) [file ppat.1001099.s001.tif]

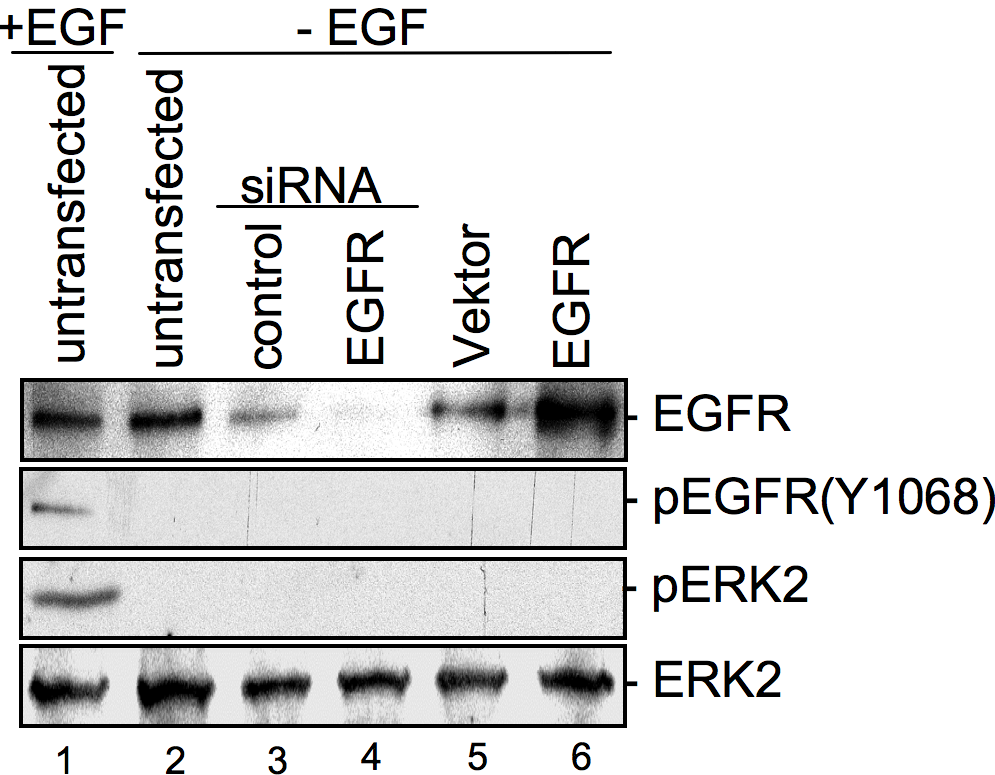

Supplement: Figure S2 — Modulation of the overall EGFR number does not alter the basal EGFR-signaling. A549 cells were left untransfected (lane 1, 2) or were transfected with siRNA (lane 3, 4) for 48 h or expression constructs (lane 5, 6) for 24 h hours and subsequently lysed. Lysates of untransfected cells, which were stimulated with EGF (30 ng/ml, 10 min) served as positive control (lane 1). WBs were probed with the indicated antibodies. (0.36 MB TIF) [file ppat.1001099.s002.tif]

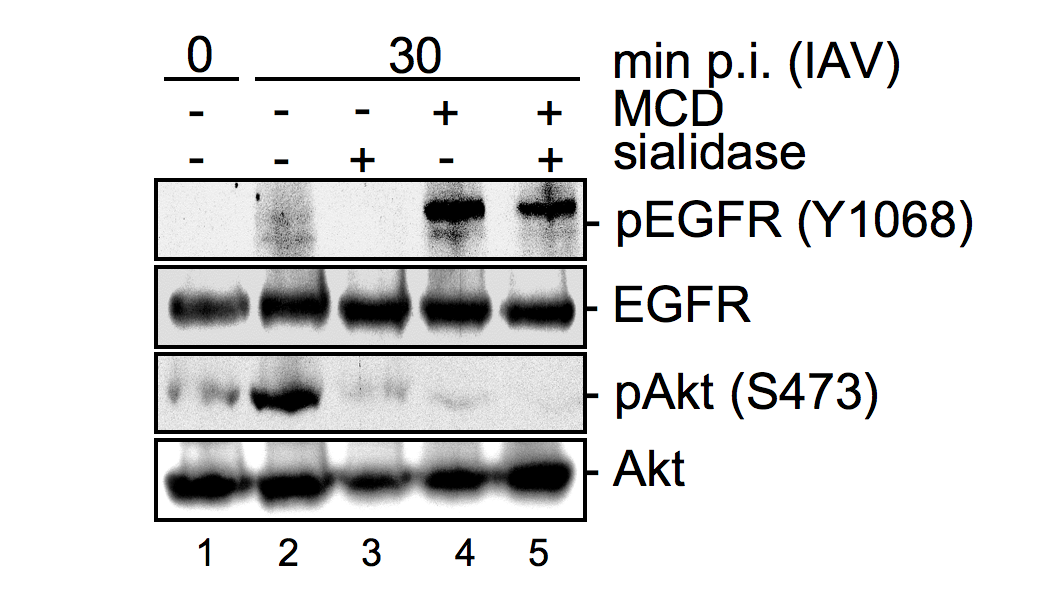

Supplement: Figure S3 — Cholesterol depletion inhibits IAV-induced EGFR downstream signaling. A549 cells were left untreated or were treated with sialidase (0.01 units/ml, 3 h, 37°C). After 2 h incubation with sialidase, MCD (40 µg/ml) was added in one untreated and one sialidase treated probe. After incubation for another hour at 37°C, PR8 (MOI = 100) was attached at 4°C for 60 min and subsequently incubated for further 30 min at 37°C, before cell-lysis. WBs were probed with the indicated antibodies. (0.23 MB TIF) [file ppat.1001099.s003.tif]

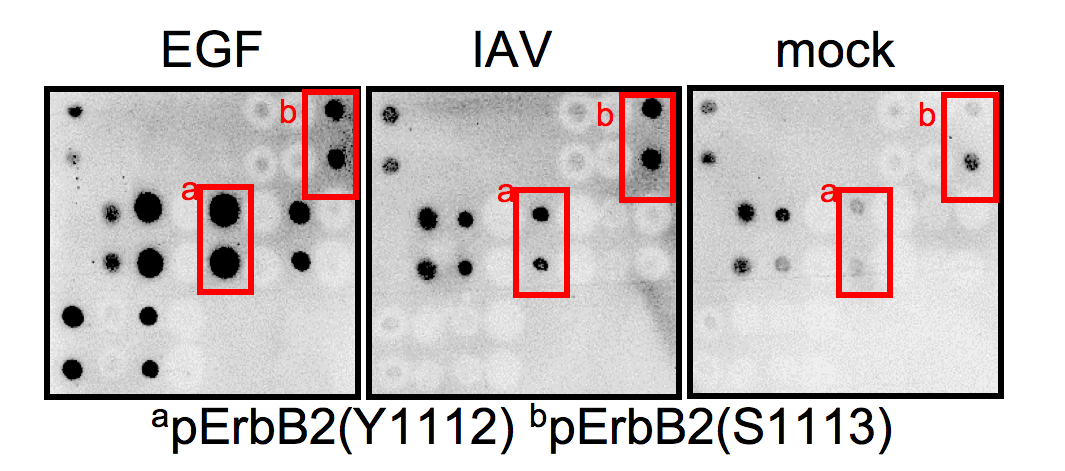

Supplement: Figure S4 — Screening for additional EGF or virus-induced phosphorylation sites in EGFR family members. A549 cells were either left untreated or incubated with EGF (30 ng/ml) or infected with IAV strain PR8 (MOI = 100) for 15 min at 37°C. Cell lysates were analysed for different phosphosites in different EGFR-family members, using the Human EGFR Phosphorylation Antibody Array 1 from RayBio (see material and methods). (0.36 MB TIF) [file ppat.1001099.s004.tif]
